# Improving adherence to an online intervention for low mood with a virtual coach: study protocol of a pilot randomized controlled trial

**DOI:** 10.1186/s13063-020-04777-2

**Published:** 2020-10-16

**Authors:** Simon Provoost, Annet Kleiboer, José Ornelas, Tibor Bosse, Jeroen Ruwaard, Artur Rocha, Pim Cuijpers, Heleen Riper

**Affiliations:** 1 Department of Clinical, Neuro- and Developmental Psychology, Clinical Psychology Section, VU University and Amsterdam Public Health Research Institute, Amsterdam, Netherlands; 2 Institute for Systems and Computer Engineering, Technology and Science, Porto, Portugal; 3 Behavioural Science Institute, Radboud University, Nijmegen, Netherlands; 4 Department of Psychiatry, Amsterdam UMC, Location VU University Medical Centre, and Amsterdam Public Health Research Institute, Amsterdam, Netherlands; 5 GGZ inGeest Specialized Mental Health Care, Amsterdam, Netherlands

**Keywords:** iCBT, Study protocol, Pilot RCT, Adherence, Virtual coach, Automated support, Low mood

## Abstract

**Background:**

Internet-based cognitive-behavioral therapy (iCBT) is more effective when it is guided by human support than when it is unguided. This may be attributable to higher adherence rates that result from a positive effect of the accompanying support on motivation and on engagement with the intervention. This protocol presents the design of a pilot randomized controlled trial that aims to start bridging the gap between guided and unguided interventions. It will test an intervention that includes automated support delivered by an embodied conversational agent (ECA) in the form of a virtual coach.

**Methods/design:**

The study will employ a pilot two-armed randomized controlled trial design. The primary outcomes of the trial will be (1) the effectiveness of iCBT, as supported by a virtual coach, in terms of improved intervention adherence in comparison with unguided iCBT, and (2) the feasibility of a future, larger-scale trial in terms of recruitment, acceptability, and sample size calculation. Secondary aims will be to assess the virtual coach’s effect on motivation, users’ perceptions of the virtual coach, and general feasibility of the intervention as supported by a virtual coach. We will recruit *N* = 70 participants from the general population who wish to learn how they can improve their mood by using Moodbuster Lite, a 4-week cognitive-behavioral therapy course. Candidates with symptoms of moderate to severe depression will be excluded from study participation. Included participants will be randomized in a 1:1 ratio to either (1) Moodbuster Lite with automated support delivered by a virtual coach or (2) Moodbuster Lite without automated support. Assessments will be taken at baseline and post-study 4 weeks later.

**Discussion:**

The study will assess the preliminary effectiveness of a virtual coach in improving adherence and will determine the feasibility of a larger-scale RCT. It could represent a significant step in bridging the gap between guided and unguided iCBT interventions.

**Trial registration:**

Netherlands Trial Register (NTR) NL8110. Registered on 23 October 2019.

## Background

The most widely studied online interventions for depression are those based on cognitive-behavioral therapy (CBT) [1]. Such interventions may be guided or unguided. Guided interventions typically include regular feedback and support by professional health care workers, licensed therapists, or trained volunteers, either via secured email exchange or via messaging systems within the intervention platforms. In shorter interventions, mostly up to eight sessions, support often takes the form of coaching, but in more intensive types of treatment, it may be more therapeutic in nature. Guided interventions have been found more effective in terms of symptom improvement [2–5]. That may be explained by a more positive effect of the guidance on motivation and engagement, and hence on adherence rates [6, 7]. However, as guided interventions require the involvement of supportive humans, unguided interventions are potentially more scalable, more accessible, and less expensive [8]. This study is part of a project to bridge the gap between guided and unguided self-help internet-based CBT (iCBT) interventions for depression, using *embodied conversational agents* (ECAs) to automate coaching support. ECAs are more or less autonomous and intelligent software entities with a graphical embodiment. They are used to communicate with the user [9].

The idea of using ECAs in psychological treatment procedures goes back roughly a decade [10], and a recent scoping review has shown that many different such applications have since been developed for a variety of common mental health disorders [11]. In the context of depression, ECAs have been proposed for a broad range of applications. For example, ECAs have taken on the role of an interviewer that engages in face-to-face interaction with users to make them feel more comfortable in talking about and sharing distressing information [12], or the role of a virtual nurse who guides hospital patients with depression through their discharge procedure [13], or that of an empathic therapist who helps people navigate the Beck Depression Inventory questionnaire [14]. A number of studies have applied ECAs in the context of an iCBT for depression. Study designs varied widely. Martínez-Miranda and colleagues conducted a pilot study in which an ECA supported users throughout a CBT intervention [15]. Their evaluation, involving *N* = 8 adult participants with mild to moderate depression, focused primarily on the feasibility of the cognitive change model employed by the ECA to regulate its own emotional responses, for example by providing more empathic feedback or facial expressions. In a randomized controlled study by Kelders of an online acceptance and commitment therapy involving *N* = 134 adults with mild to moderate depression, half of the participants received automated feedback accompanied by a picture of a clinician and the other half received human support [7]. The study concluded that, although participants receiving human support were more involved in the intervention than those receiving automated feedback (as scored on the Personal Involvement Inventory [16]), they were not significantly more adherent in terms of intervention completion. A pilot study by Ring and colleagues aimed to create a one-on-one therapeutic conversation with a virtual counselor [17]. In a pre–post-test study design including *N* = 10 participants with mild to moderate depression, most users reported that the agent understood their emotions, but no significant improvements in depressive symptoms were found. Another pre–post-test pilot study investigated the acceptability and usability of a user-adapted, ECA-supported interactive platform addressing depression and suicide symptoms in a convenience sample of *N* = 60 participants [18]. It concluded that system usability and the acceptability of the agent’s emotional responses were sufficient for the researchers to continue preparing the system for an initial clinical trial. A study by Fitzpatrick and colleagues looked at the feasibility, acceptability, and preliminary effectiveness of a conversational agent called Woebot, which delivered CBT-based self-help content in a text-based conversational format [19]; *N* = 70 university students who self-identified as experiencing depression and anxiety symptoms were randomized to using Woebot or to reading a book on depression. The intervention group reported significant reductions in depressive symptoms compared with the control group (*d* = 0.44). In another study, Suganuma and colleagues investigated the feasibility and acceptability of an ECA-delivered CBT-based intervention that aimed to determine users’ mental and physical status in order to make appropriate behavioral suggestions. A non-clinical intervention group of *n* = 191 users was compared with *n* = 263 study participants who did not use the intervention. The intervention showed some initial effectiveness in terms of mental health improvement [20]. Many of the applications described in this paragraph were judged acceptable and feasible, and some of the studies even showed that positive treatment effects can be accomplished using ECA-based interventions (e.g., [19, 20]).

Although the studies just reviewed have shown promising results, most did not focus on ECAs in a supportive role as an adjunct to an iCBT intervention (intervention + ECA), but rather on the ECA as a medium through which iCBT could be delivered (intervention = ECA). In order to strengthen the evidence for the use of ECAs as an adjunct to improve iCBT interventions, a study would need to compare an ECA-supported intervention with the same intervention with either human support or no support. Of the studies cited above, only the one by Kelders [7] used such a design. That study, however, focused primarily on automated support through text messages, with the support embodied with a picture of a clinician. Though this does satisfy our criteria for what an ECA is, we might question how well the results generalize to interventions utilizing more sophisticated ECA technology. We aim to address this gap in the literature by comparing outcomes of participants in an existing intervention with added ECA support (our intervention group) with the outcomes of participants in the same intervention without ECA support (our control group). Our general hypothesis is that by simulating a number of human support factors—specific factors such as motivational interviewing techniques and feedback to CBT exercises and common factors such as empathic communication [21]—an ECA can positively affect motivation and engagement, and thereby adherence rates. This, in turn, may increase the clinical effectiveness of iCBT interventions in which traditional human support is unavailable [22]. Given the novelty of our approach, which combines an existing iCBT intervention with ECA support, we have opted for a pilot randomized controlled trial, whose primary aims will be to compare adherence rates between the two study groups and to assess the feasibility of a future larger-scale trial. Secondary aims include assessing within- and between-group participant motivation for performing and continuing the intervention, gauging users’ acceptance of and perceived relationship with the supportive ECA, and estimating the feasibility of the entire system in terms of user satisfaction, usability, and preliminary effectiveness.

## Methods/design

### Study design

**Fig. 1:**
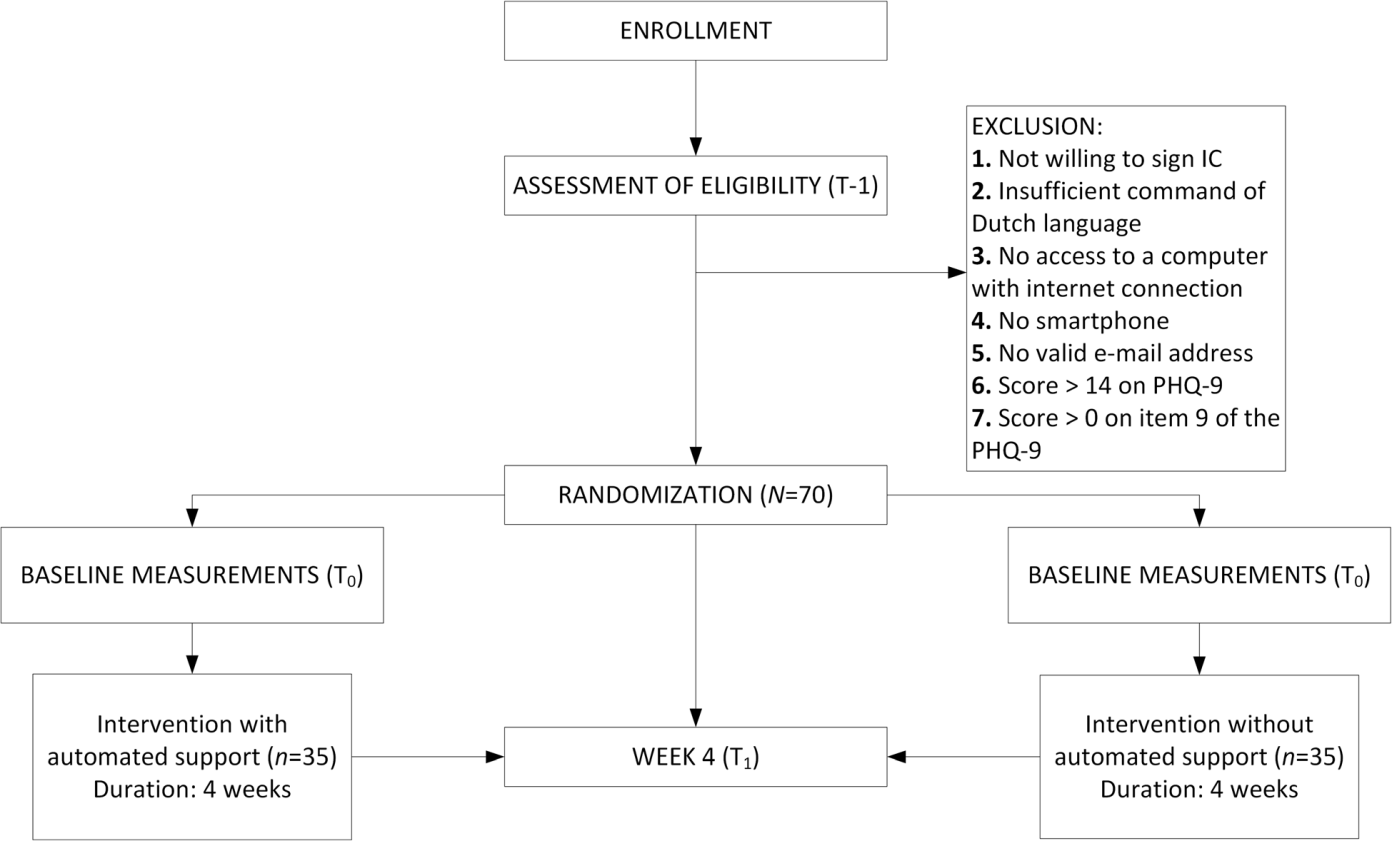
Flowchart of the study design

The study is designed as a pilot non-blinded two-armed randomized controlled trial (*N* = 70) in which people with low mood from the general population will be randomly allocated either to an intervention for improving mood with automated support delivered by a virtual coach (*n* = 35) or to the same intervention without the automated support (*n* = 35). The study protocol has been approved by the Medical Ethics Committee of the VU University Medical Centre, Amsterdam (registration number 2019.388). Written informed consent will be obtained from all participants. Figure 1 displays the flowchart of the study design in accordance with the SPIRIT guidelines [23, 24].

### Assessments

Assessments will be taken after enrollment (T−1), at baseline (T_0_), and at the end of study participation 4 weeks after baseline (T_1_). Questionnaires will be self-administered and completed online. Table 1 provides an overview of the measures employed at specific time points.

**Table 1:** Measures administered at each assessment interval

Questionnaire	Aim	Enrollment (T−1)	Allocation (T_0_)	Post-study (T_1_)
Mental health				
PHQ-9	*Screener*	X		
HADS-D	*Mental health*		X	X
Feasibility				
SUS	*System usability*			X
CSQ-I	*User satisfaction*			X
Study completion	*Reasons for non-adherence*			X
Motivation				
SMFL	*Current use*		X	X
Continued use	*Continued use*			X
Coach acceptance				
WAI-SR*	*Relationship*			X
Acceptance*	*Acceptance*			X

*Applies to the intervention group only

### Participants

#### Inclusion criteria

People from the general population in the Netherlands, aged 18 years or older, will be eligible for recruitment if they express a desire to learn how to improve their mood.

#### Exclusion criteria

Candidates will be excluded from the study if they (i) are not willing to sign the informed consent form, (ii) do not have adequate proficiency in the Dutch language, (iii) do not have a computer with internet access, (iv) do not have a smartphone, (v) do not have a valid email address, (vi) have moderate to severe depression, or (vii) are identified as at risk for suicide. The Patient Health Questionnaire-9 (PHQ-9) will be used to assess whether exclusion criteria *vi* and *vii* apply. Excluded candidates will receive an email detailing the reason for their exclusion. If exclusion criterion *vi* applies (a score of 15 or higher on the PHQ-9), they will be advised to contact their general practitioner, and if *vii* applies (a score of 1 or higher on PHQ-9 item 9), they will also be referred to a national help and crisis line for people at risk of suicide (https://www.113.nl).

### Recruitment

Participants will be recruited in an open recruitment strategy via advertisements in digital media (Facebook, Google Ads) and http://www.link2trials.com. Interested persons will be invited to express their interest in participation by filling out a web form, after which they will receive an information brochure and an informed consent form. People who sign the consent form will receive a link to the online screening questionnaire and, once found eligible for participation, will be sent final instructions and login credentials for taking part in the study. Participants will receive 30 euro if they complete the T_1_ assessments, irrespective of how much time they have committed to the course. They will be free to discontinue study participation at any time, and participation places no restrictions on their use of alternative sources of help.

### Randomization and blinding

Participants will be randomly assigned by an independent researcher to either Moodbuster Lite with automated support (intervention group) or Moodbuster Lite without automated support (control group). That will take place in a 1:1 ratio and on the basis of a computer-generated block randomization table with random block sizes [25]. Group allocation cannot be blinded to participants, because a description of the study’s research aim—improving intervention adherence with automated support by a virtual coach—must be provided in the information letter; whether or not automated feedback is provided will hence be obvious to participants. The principal investigator, who coordinates the study and conducts the data analysis, will not be blinded to the participants’ group allocation.

### Interventions

#### Moodbuster Lite

Moodbuster Lite is a 4-week therapeutic course aimed at improving mood. It is a light-weight version of the Moodbuster for Depression intervention [26, 27] and consists of a web-based and a mobile component. Compared to Moodbuster for Depression, which also contains a number of cognitive therapy-based modules, the focus of Moodbuster Lite is on behavioral activation [28]. Through activity scheduling, participants learn to turn a “negative spiral,” with few pleasant activities leading to few positive stimuli, a low mood, and little incentive to perform more activities, into a “positive spiral,” with more pleasant activities leading to more positive stimuli, a better mood, and incentive to remain active. A secure web-based platform provides access to online lessons, homework exercises, a mood graph, and a calendar. A smartphone application, designed for both Android and iOS, prompts participants three times a day with a request to rate their current mood, and an overview of the participant’s responses is shown in both the app and the web platform’s mood graph. The course consists of three lessons that were adapted from the Moodbuster for Depression intervention to fit the low-mood context of this study: (1) Introduction, (2) Psychoeducation, and (3) Pleasant activities. The first lesson has also been extended with some exercises based on motivational interviewing [29] to increase participants’ motivation for completing the course. For the purpose of the current study, an optional virtual coach has been embedded into the platform to provide automated support at the beginning and the end of every lesson and halfway through lesson 3, the longest lesson. For this study, participants are advised, but not obliged, to complete the intervention in a time span of 4 weeks. On completion, participants retain their access to the platform for about another 5 months. An overview of the intervention is shown in Table 2.

**Table 2:** Overview of the Moodbuster Lite course as used in this pilot RCT

	Lesson 1	Lesson 2	Lesson 3
**Topic**	Introduction	Psychoeducation	Pleasant activities
**Time frame**	Week 1	Week 1	Weeks 2–4
**Length**	22 pages	11 pages	17 pages
**Conversations with virtual coach**	2	1–2	2–3

#### Automated support

##### Technical implementation

**Fig. 2:**
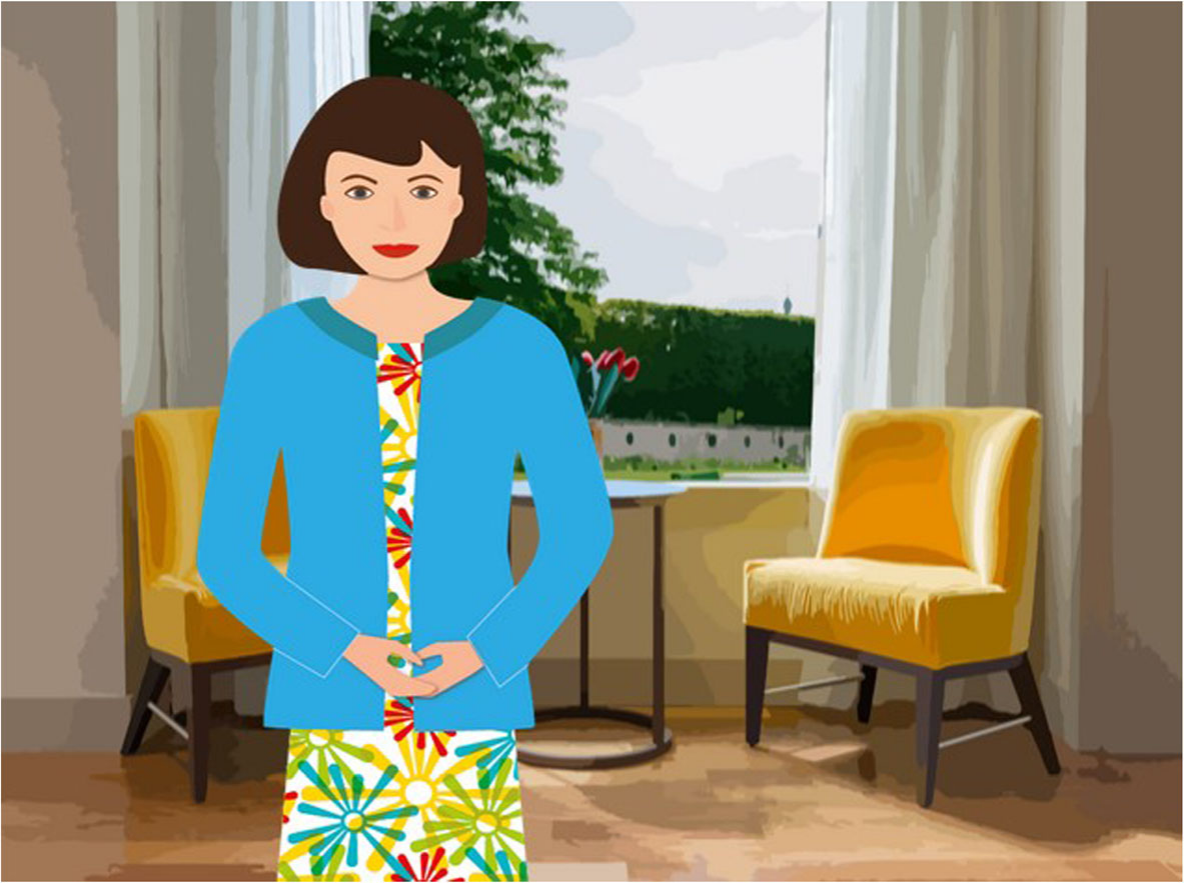
The virtual coach embedded in the Moodbuster Lite platform

Automated support is delivered by a virtual coach in the form of an ECA. The ECA has been implemented in TyranoBuilder [30], a JavaScript-based software package for the development of visual novels that can be used to implement text-based dialogues with a virtual character. Our choice for TyranoBuilder was strongly motivated by the fact that applications can be exported in a browser format that allows them to be embedded in web pages (Fig. 2).

##### Embodiment

We have embodied the ECA using a single two-dimensional static cartoon-like character, taking into account the following recommendations from the literature on ECAs for motivational and coaching purposes. We have opted for a cartoon-like embodiment, as increased realism is not that important for involvement, distance, and use intentions, and may even set high expectations that the ECA cannot meet [31]. With regard to gender, we have chosen a female embodiment, as that is what people on average prefer [32]. The ECA is endowed with a number of facial expressions (friendly, smiling, compassionate, questioning; see Additional file 1), such that it can convey a sense of empathy [33]; we have not given the ECA negative facial expressions [34]. Finally, the ECA is designed to look as if it could be part of a therapy team, increasing its credibility by giving it a semi-formal friendly appearance and placing it before a background reminiscent of a therapy office [35].

##### Conversations

The conversations have been designed in collaboration with a licensed therapist and are based on guidelines for e-coaching [35] and principles of motivational interviewing [29]. Some examples of guidelines for providing feedback we have applied are to (1) use correct greetings and closings; (2) use communication skills such as beginning a message with a compliment; (3) structure feedback, for example by not giving feedback on more than two subjects; (4) refer to things the participants have done, such as completing exercises or recording their moods; and (5) keep text readable by using short, clear sentences. With regard to the motivational interviewing, we have focused on increasing an individual’s willingness to change behavior, as well as on their confidence in their ability to do so, both of which are important for being “ready” to change. Baseline values of a participant’s willingness to change and confidence in their ability to do so are established using the *importance and confidence ruler* exercises in lesson 1 of the intervention. If importance or confidence is low, the virtual coach presents specific exercises aimed either at increasing the discrepancy between a participant’s goals and their current behavior and emphasizing the importance of change, or at enhancing a participant’s self-efficacy and emphasizing confidence in their ability to change. These elements have been incorporated into all the conversations except the introductory and final ones, thus providing us with the general conversation structure shown in Table 3. Conversations after each lesson always take place, focused on providing feedback, while conversations before a lesson take place only if motivation is considered low or if the previous lesson received a negative evaluation. Such evaluations can be given by free text input at the end of each lesson, and a sentiment analysis algorithm [36] is used to determine its valence (negative or positive).

**Table 3:** The differential stages in the conversations

Stage	Before the lesson	After the lesson
1	Greeting	Greeting
2	Compliment or positive note	Compliment or positive note
3	Reflection on evaluation of previous lesson	Reflection on current lesson
4	Re-evaluation of confidence or willingness	Confidence or importance work
5	Reference to current lesson	Reference to next lesson
6	Goodbye	Goodbye

##### Conversation trees

**Fig. 3:**
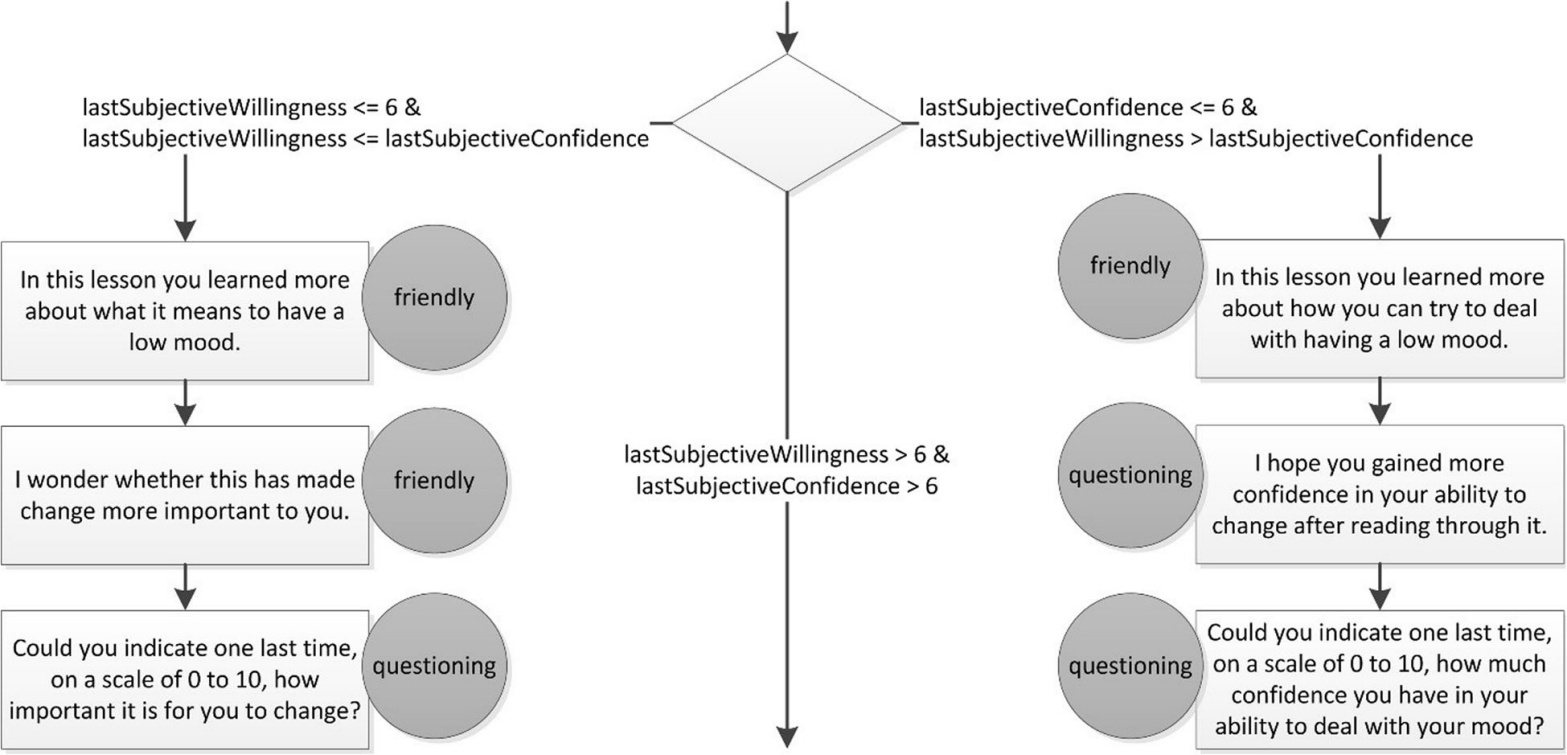
A conversation tree snippet from the dialogue that takes place after the second lesson

The conversations take place through text-based messages appearing beneath the virtual coach (see Fig. 2), and the user can proceed through the conversations by clicking the mouse button, or now and then by selecting or providing an answer when asked a question. Although much progress is currently being made in speech and natural language processing, we decided to represent our dialogues in textual conversation trees for several reasons: (1) speech and natural language processing are still far from flawless; (2) automatic interpretation and accurate response to semantic content are difficult; (3) conversation trees can be more easily interpreted by domain experts such as clinical psychologists; (4) conversation trees are deterministic, meaning that there is an exhaustive set of possible conversations that can be checked for inconsistencies; and (5) certain paths through the tree can be made conditional, for example based on an answer to an earlier question in the lesson or conversation, thus enabling conversations to be personalized. For illustrative purposes, Fig. 3 shows an excerpt from one of the conversation trees. The diamond represents a decision point in the conversation tree, rectangles represent utterances by the virtual coach, and circles indicate the corresponding facial expressions. The excerpt compares the latest confidence and willingness ratings provided by the user. If both values are higher than 6, the confidence and importance work is skipped. If one value is 6 or lower, the user is asked to re-evaluate the lower rating, prioritizing willingness over confidence, after which the tree continues with a suitable exercise. Additional file 2 provides additional information about the variables used in this excerpt.

### Trial organization

The study is run from VU University Amsterdam, with no other study centers participating. The principal investigator is responsible for coordinating the study, which includes the recruitment of participants and the informed consent procedure, responding to questions and requests from (potential) participants, providing participants with access to the study materials, monitoring participants throughout the study, handling participant reimbursements, data collection, and reporting on the progress of the study to the steering committee members and medical ethical committee. The steering committee (see title page for members) agreed on the final version of this protocol and is responsible for reviewing the progress of the study, and for agreeing on changes to the protocol or study materials, if necessary, to keep the study running properly. Meetings of the steering committee are scheduled when necessary. The trial management committee is composed of the principal investigator and project leader. It is responsible for the study planning, organization of steering committee meetings, reporting to the medical ethical committee of study progress, maintenance of the trial master file, budget administration, and data verification. The trial management committee meets on a monthly basis. An IT team is responsible for the maintenance of the intervention platform and data collection from the platform. As this is a relatively small pilot study, there is no Stakeholder and Public Involvement Group.

Earlier large-scale research using the Moodbuster platform did not result in any known serious adverse events (SAEs) or serious adverse device events (SADEs). If SAEs or SADEs do occur, they will be discussed in the research team and reported to the Dutch Health and Youth Care Inspectorate. Any other adverse events reported spontaneously by the participants or observed by the investigators will be recorded. Due to the low-risk nature of the study, there is no anticipated harm and compensation for trial participation. Participants can contact an independent researcher if they run into issues during the study, and a licensed psychiatrist can be consulted in case issues of a medical or mental health related nature arise.

Significant amendments to the study protocol will be communicated to the medical ethical committee that approved the study, and an update will be made to the study information in the Dutch Trial Registry. Results will be published in a peer-reviewed journal and reported to the medical ethical committee that approved the study.

### Primary outcome measures

#### Adherence

The primary outcome measure will be intervention adherence. According to the definition we have adopted, “adherence” describes the extent to which individuals are exposed to the content of the intervention [37]. Previously, this has been operationalized by dividing the number of completed sessions or modules by the maximum number [38], but because our 3-lesson course is relatively short, we will use the completed and maximum numbers of pages that make up the lessons. Including conversations with the coach, lesson 1 has 22 pages (20 in the control condition), lesson 2 has 13 (11 in the control condition), and lesson 3 has 20 pages (17 in the control condition). As a secondary way of measuring adherence, we will look at the *ecological momentary assessment* of mood via the smartphone application, whereby (similarly to adherence to the intervention content) we will operationalize adherence as the number of mood assessments made divided by the maximum possible number. There will be three mood assessments every day, meaning that participants can answer a maximum of 84 mood rating requests during the 4 weeks of the study.

### Secondary outcome measures

#### Motivation

Motivation for taking part in the intervention will be assessed in both groups by the Short Motivation Feedback List (SMFL) [39]. It consists of eight 10-point Likert-scale items ranging from “completely disagree” to “completely agree,” designed to capture the level and type (external, introjected, or identified) of a patient’s treatment motivation. The SMFL is based on self-determination theory [40] and has been found to have a congeneric reliability ranging from 0.81 to 0.93 [39]. There are two different versions. The pre-intervention version will be assessed at baseline (T_0_) and the post-intervention version after 4 weeks (T_1_). Motivation to continue using the intervention will be assessed by a single statement, “I intend to continue using the platform to schedule and perform activities,” assessed on a 5-point Likert scale ranging from “completely disagree” to “completely agree.”

#### Relationship with the coach

After study completion (T_1_), participants in the intervention group will assess their relationship with the virtual coach on the Bond scale of the Revised Short Version of the Working Alliance Inventory (WAI-SR) [41, 42]. The WAI-SR rates the quality of the therapeutic relationship with the virtual coach, and it has been adjusted to our context by replacing the name of the therapist with the word “coach.” The Bond scale consists of four 5-point Likert-scale items ranging from 1 (seldom) to 5 (always). The final raw score may range from 4 to 20, with higher scores indicating a better bond between participant and coach. The psychometric properties of the questionnaire are satisfactory [42].

#### Acceptance of the coach

Acceptance of the virtual coach will be assessed in the intervention group after 4 weeks (T_1_) using a set of six 7-point Likert-scale items. This scale has been previously used to measure attitudes toward a virtual discharge nurse [13] and has been adjusted to our context of iCBT. An overview of the items is provided in Table 4. Participants are asked to elaborate on their answers to each of these questions in an open text format.

**Table 4:** Self-report measures of attitudes toward the virtual coach

Measure	Question	Likert-scale extremes
Satisfaction	How satisfied were you with the virtual coach?	Not at all–Very satisfied
Usability	How easy was it talking to the virtual coach?	Easy–Difficult
Continue	How much would you like to continue working with the virtual coach if the course continued?	Not at all–Very much
Relationship	How would you characterize your relationship with the virtual coach?	Complete stranger–Close friend
Preference	Would you rather have followed the course with or without the virtual coach?	Definitely prefer no coach–Definitely prefer virtual coach
Adherence	How likely is it that you will follow the virtual coach’s advice?	Not at all likely–Very likely

#### System usability

Usability of the platform will be assessed after week 4 (T_1_) by the System Usability Scale (SUS) [43]. The SUS is composed of ten 5-point Likert-scale items with response options ranging from 0 (strongly disagree) to 4 (strongly agree). Total scores are converted to a scale ranging from 0 to 100, where higher scores are indicative of higher platform usability. The SUS is considered a reliable instrument, and scores higher than 68 indicate “good” usability [44].

#### User satisfaction

User satisfaction with the web-based intervention will be assessed by the Client Satisfaction Questionnaire for internet-based interventions (CSQ-I) [45], an adaptation of the original CSQ [46]. The CSQ-I is composed of eight 4-point Likert-scale items with response options ranging from “does not apply to me” to “applies to me.” Total scores range from 8 to 32, with higher scores indicating greater client satisfaction. The CSQ-I has been found to be a reliable instrument [45].

#### Mental health status

Mental health status will be assessed using the Depression subscale of the Hospital Anxiety and Depression Scale (HADS-D) [47], consisting of seven items, each assessed on a 3-point scale. Total scores range from 0 to 21, and higher scores indicate more severe depression symptoms. An often used cutoff score for the HADS-D is 8 or higher, standing for “relevant symptoms of depression.” The HADS has been shown to be a reliable and valid instrument in various populations [48].

#### Mood

Participants’ mood will be assessed through ecological momentary assessments on a smartphone application that works on both Android and iOS systems. The application prompts participants three times a day to rate their mood on a scale of 1 to 7 (see Additional file 3).

#### Reasons for non-adherence

At the end of the study, at T_1_, participants will be asked online whether they completed the intervention and used it for the full duration of the study. If their response is negative, they will be asked to provide a rationale for not having completed the intervention or the study.

#### Level of engagement with the intervention

The third lesson is designed to stimulate users to schedule, perform, and evaluate pleasant activities. The number of these activities over time is assessed through log file analysis. Whether participants keep scheduling and recording activities for the duration of the study is an indicator of their engagement with the course, and of whether it has managed to make them more active.

#### Other measures

Screening for mental health issues will be performed before group allocation (T−1) using the Patient Health Questionnaire-9 (PHQ-9) [49], in order to deter people with more severe issues from taking part in the study. The PHQ-9 is composed of nine statements, each scored on a scale of 0 (not at all) to 3 (almost every day). Total scores range from 0 to 27, with higher scores indicating more severe depression, and scores over 14 moderate to severe depression (see the “Exclusion criteria” section above). The PHQ-9 is considered to have good psychometric properties [50].

### Sample size

Since this study is a first in its sort, we know of no literature that indicates what effect size could be expected. Following the recommendation of Teare and colleagues [51], we plan to recruit 70 participants to determine the group means and standard deviations required for an estimation of the effect and sample sizes in a future RCT.

### Statistical analysis

#### Primary analysis

The primary analysis will focus on the preliminary effectiveness of the virtual agent with respect to intervention adherence, as assessed in terms of intervention completion and mood recording response rates. Intervention completion will be assessed by calculating point estimates with corresponding 95% confidence intervals for both the intervention and the control group; a general linear model will be used to estimate the preliminary effect at the alpha < 0.05 significance level. That information will enable us to calculate the sample size required for observing a similar intervention effect in a larger RCT. To assess the mood recording response rate, we will conduct a logistic mixed-effects analysis to determine variations in adherence over time, following a similar analysis we performed in a previous ecological momentary assessment study [52].

#### Secondary analysis

All secondary study parameters will be assessed with descriptive analysis, with formal tests merely serving to gain an estimation of possible group differences. Group differences will all be represented by point estimates and 95% confidence intervals. Within-group changes (pre–post, T_0_–T_1_) in motivation for taking part in the intervention (on the SMFL) and in mental health status (HADS-D) will also be tested formally with a mixed-effects model to estimate a time × group interaction effect and individual differences. Additionally, usability (SUS) and user satisfaction (CSQ-I) scores will be compared with the established benchmarks. Mood as measured by the smartphone records, and scheduled and recorded activities as measured in the platform, will only be analyzed descriptively. No subgroup analyses will be performed.

#### Data management

On the informed consent form, participants will be asked if they agree to the use of their data in future research on the same topic at VU University, and to their data being shared with regulatory authorities when required. This trial does not involve the collection of biological specimens for storage. All raw data will be stored on a secure local server at the VU University in Amsterdam, which is regularly backed up. Paper-based documents will be stored in a keycard-secured archive at the Department of Clinical, Neuro- and Developmental Psychology. All participants will be de-identified upon randomization by linking their participant number to a random study participant code. In the study, participants will be referred to exclusively by that participant code, and the document linking the two numbers will be destroyed once the study is over and results have been disseminated. Because this study is relatively small and investigator-initiated, no data monitoring committee or auditing process is required. Because we do not expect serious negative outcomes for the participants, we do not conduct an interim analysis and there are no subsequent formal stopping rules.

## Discussion

The study described in this protocol paper is a pilot randomized controlled trial that will compare an unguided intervention for low mood with the same intervention with additional automated guidance provided in the form of a virtual coach. The main goal is to gain an estimate of the effectiveness of the virtual coach in terms of improving adherence to the intervention. That will help determine the feasibility and necessity of a future larger-scale trial.

Many studies have shown that online interventions that include human guidance are generally more effective than ones that do not. Human therapists or coaches that can provide such guidance are not always available, however, and the time of trained therapists is especially costly. Existing rules and protocols about providing guidance can be programmed into the interventions themselves so as to be automatically safeguarded. Moreover, automated support through ECAs enables human support factors such as empathy to be delivered more effectively. Automated support could improve adherence rates of guided, and especially of unguided, web-based interventions and could thus improve their effectiveness.

While ECAs have been shown in many studies to be a feasible and acceptable technology in the domain of clinical psychology, very few applications have so far moved beyond the piloting phase. That is also the case for ECAs in iCBT contexts, where studies up to now have either been underpowered, have lacked control groups that set apart the ECA as the active ingredient, or have lacked depth in terms of underlying ECA technology. This study addresses these gaps in the literature in the following ways: (1) we designed a virtual coach that delivers automated support to iCBT for low mood, (2) we embedded it in an existing platform so that the platform can be used either with or without the ECA, and (3) we will estimate the effectiveness of a virtual coach in improving adherence and determine the parameters required for a proper RCT sample size calculation. Despite the technical limitations that come with embedding an ECA in an existing intervention platform, our virtual coach satisfies the criteria for an ECA—graphical embodiment, communicating with the user, and applying a form of reasoning—and conforms to recommendations from the literature. As a result, this study could represent a significant step in bridging the gap between guided and unguided iCBT interventions.

## Trial status

### Protocol version

Version 1.0, 25 October 2019

### Recruitment

Start date: 1 January 2021

End date: 30 June 2021

## Supplementary information

**Additional file 1.** The four different expressions of the virtual coach: friendly, smiling, compassionate, questioning (left to right).

**Additional file 2.** Additional information about the variables used in the conversation tree excerpt depicted in Fig. 3.

**Additional file 3.** Screenshot of the Moodbuster smartphone application.

## Data Availability

Anonymized data used for statistical analysis will be published with the results paper and archived in a public data repository. Study materials such as the intervention content and informed consent form will be shared with other researchers upon reasonable request.
