# Supplementary material for: Improving adherence to an online intervention for low mood with a virtual coach: study protocol of a pilot randomized controlled trial
Source: Trials. 2020 Oct 16;21:860. doi: 10.1186/s13063-020-04777-2 (PMC7565359; doi:10.1186/s13063-020-04777-2)
Supplement: Supplementary file 1 — Additional file 1. The four different expressions of the virtual coach: friendly, smiling, compassionate, questioning (left to right). [file 13063_2020_4777_MOESM1_ESM.docx]

**Appendix 1**


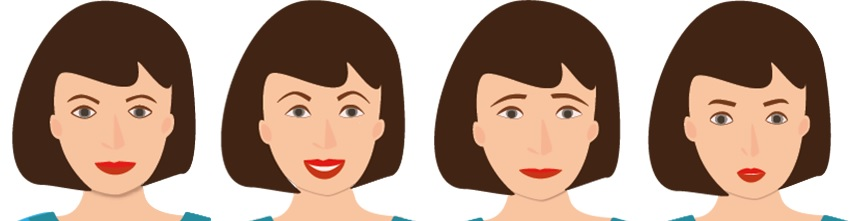


The four different expressions of the virtual coach: friendly, smiling, compassionate, questioning (left

to right)
