# Supplementary material for: Improving adherence to an online intervention for low mood with a virtual coach: study protocol of a pilot randomized controlled trial
Source: Trials. 2020 Oct 16;21:860. doi: 10.1186/s13063-020-04777-2 (PMC7565359; doi:10.1186/s13063-020-04777-2)
Supplement: Supplementary file 2 — Additional file 2. Additional information about the variables used in the conversation tree excerpt depicted in Fig. 3. [file 13063_2020_4777_MOESM2_ESM.docx]

**Appendix 2**

Additional information about the variables used in the conversation tree excerpt depicted in Figure 3

| **Variable** | **Origin** | **Use** |
| --- | --- | --- |
| name: lastSubjectiveWillingness  value range: 0–10 | The latest “willingness to change” rating provided by the user, during either the first lesson or a subsequent conversation | Determines whether importance work is needed, and is compared with the confidence rating to determine which of the two to prioritize |
| name: lastSubjectiveConfidence  value range: 0–10 | The latest “confidence in ability to change” rating provided by the user, during either the first lesson or a subsequent conversation | Determines whether confidence work is needed, and is compared with the willingness rating to determine which of the two to prioritize |
