# Supplementary figures and images for: Improving adherence to an online intervention for low mood with a virtual coach: study protocol of a pilot randomized controlled trial
Source: Trials. 2020 Oct 16;21:860. doi: 10.1186/s13063-020-04777-2 (PMC7565359; doi:10.1186/s13063-020-04777-2)

**Appendix 3**


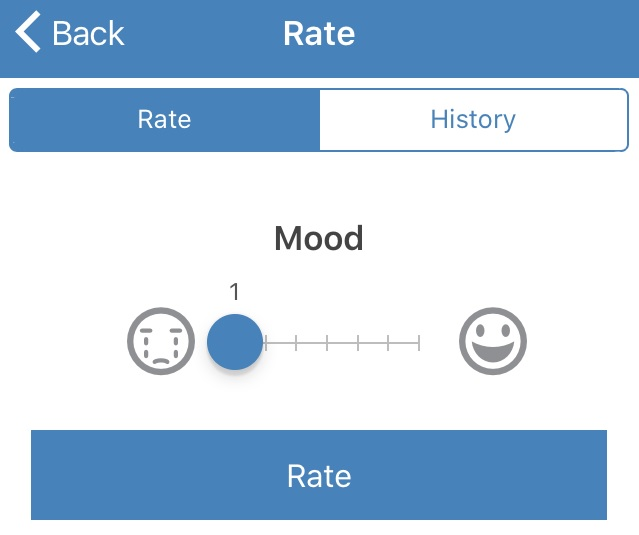


Screenshot of the Moodbuster smartphone application

Supplement: Supplementary file 3 — Additional file 3. Screenshot of the Moodbuster smartphone application. [file 13063_2020_4777_MOESM3_ESM.docx]
